# *Triatoma rubrofasciata* as a potential vector for bartonellosis

**DOI:** 10.1080/22221751.2025.2494291

**Published:** 2025-04-15

**Authors:** Peichao Deng, Binglian Qin, Anli Liang, Qingan Zhou, Xiaoyin Fu, Xiaoquan Liu, Chenghui Lao, Xiaoqin Li, Shanshan He, Lili Tang, Ziwen Zhao, Wenjie Chen, Dengyu Liu, Yanwen Li, Yunliang Shi

**Affiliations:** a Parasitology Department, School of Basic Medical Sciences, Guangxi Medical University, Nanning, People’s Republic of China; b College of Animal Science and Technology, Guangxi Agricultural Engineering Vocational and Technical College, Nanning, People’s Republic of China; c Department of Livestock Disease Diagnosis, Animal Disease Prevention and Control Center of Guangxi Zhuang Autonomous Region, Nanning, People’s Republic of China; d Laboratory Department, Changle Town Health Center, Beihai, People’s Republic of China; e Key Laboratory of Basic Research on Regional Diseases (Guangxi Medical University), Education Department of Guangxi Zhuang Autonomous Region, Nanning, People’s Republic of China

**Keywords:** Human bartonellosis, Bartonella spp, triatoma rubrofasciata, transmission vector, transmission

## Abstract

*Bartonella* spp. are most often transmitted by arthropod vectors or animal bites and scratches. However, the vector species involved in the transmission of human bartonellosis remain poorly understood. This study investigated the presence of *Bartonella* in *Triatoma rubrofasciata* from Guangxi and Hainan provinces in China, evaluating its potential as a vector. *Bartonella* was identified in *T. rubrofasciata* samples through PCR amplification and sequencing of the *ITS*, *gltA*, and *rpoB* genes. The survival duration of *Bartonella* in triatomines, along with the potential for transovarial transmission was examined. Transmission experiments were conducted to determine whether *T. rubrofasciata* could transmit *Bartonella* to mice. Additionally, *Bartonella spp.* were also compared across rats, ticks, and cat fleas collected from the same regions. Results: Six *Bartonella* species were identified in *T. rubrofasciata*, including *B. rochalimae*, *B. elizabethae*, *B. tribocorum*, *B. queenslandensis*, *B. silvatica*, and *B. coopersplainsensis*. And the first three species are zoonotic. *B. rochalimae* and *B. elizabethae* were able to persist in *T. rubrofasciata* for at least eight weeks, although transovarial transmission of them was not observed. In comparison to rats, ticks, and cat fleas, *T. rubrofasciata* exhibited a higher diversity of *Bartonella* species. Laboratory experiments confirmed that *B. elizabethae* can infect mice through *T. rubrofasciata* bites or intraperitoneal injection of *T. rubrofasciata* feces. This study supports the hypothesis that *T. rubrofasciata* may serve as a vector for bartonellosis. These results broaden the current understanding of *Bartonella* transmission dynamics and highlight the potential role of triatomines in the spread of this disease.

## Introduction

*Bartonella spp.*, gram-negative intracellular bacteria, infect a broad spectrum of mammalian hosts, including humans [1], domestic animals, and wildlife [2]. Transmission occurs mainly via arthropod vectors or animal bites and scratches. Bartonellosis, an emerging infectious disease, exhibits varying morbidity globally. It particularly affects immunocompromised individuals and those in specific endemic areas. In the United States, cat-scratch disease (CSD) alone leads to approximately 12,000 outpatient visits and 500 hospitalizations annually [3]. Approximately 50 *Bartonella* species have been identified, 18 of which are pathogenic to humans [4-6]. These bacteria induce intracellular infections in human and mammalian erythrocytes [7]. As an emerging zoonotic pathogen, *Bartonella* is responsible for a variety of diseases with complex clinical presentations [8]. Key human pathogens include *B. quintana*, *B. henselae*, and *B. bacilliformis*, which are associated with trench fever, CSD, and Carrion’s disease, respectively [9]. *Bartonella quintana* infection may manifest as either an acute febrile illness or infective endocarditis [10]. *Bartonella henselae* affects the lymph nodes draining the area where a cat scratch or bite occurs, causing regional lymphadenopathy [11]. In healthy patients, symptoms typically resolve on their own or with minimal care [12]. However, in immunocompromised patients may develop serious issues such as endocarditis, encephalitis, bacillary angiomatosis, or neuroretinitis [13-15]. *Bartonella bacilliformis*, the pathogen responsible for Carrión's disease or Oroya fever, infects human erythrocytes, leading to acute hemolytic anemia (Oroya fever), followed by a chronic phase marked by cutaneous vascular eruptions similar to bacillary angiomatosis [16]. Other species, including *B. elizabethae, B. rochalimae*, *B. tribocorum*, *B. clarridgeiae*, *B. alsatica* and *B. grahamii*, are also capable of human infection [17-22], but their prevalence is limited. Although certain *Bartonella* infections may resolve spontaneously, the bacteria can evade immune detection, persist within the host, and pose significant risks if left untreated, emphasizing the necessity of timely antibiotic therapy [23].

Many *Bartonella* species are transmitted through arthropod vectors, including fleas [3], lice [24], sandflies [25], mites [26], mosquitoes [27], and ticks [28]. However, the primary routes of transmission may vary depending on the specific *Bartonella* species and their interactions with hosts. *Bartonella quintana* is primarily transmitted to humans through human body lice (*Pediculus humanus corporis*), with the bacteria from lice feces entering the bloodstream via breaks in the skin [24]. *Bartonella bacilliformis* is transmitted to humans through the bites of infected female sand flies (*Lutzomyia spp.*) [29,30]*.* In contrast, *B. henselae* is typically transmitted to humans via bites or scratches from infected cats, cat fleas (*Ctenocephalides felis*) serve as the vector for transmitting the bacterium among feline hosts [31-33]. However, the specific vectors and transmission routes for human bartonellosis, including species such as *B. rochalimae*, *B. elizabethae*, and *B. tribocorum*, remain poorly understood. This uncertainty surrounding the transmission pathways hinders effective prevention and control measures for the disease. Clarifying how different vectors transmit *Bartonella* to humans is essential. Triatomines, vectors of *Trypanosoma cruzi*, have also been implicated in the transmission of *Bartonella spp* [34,35]. For example, *Bartonella* DNA was detected in *Eratyrus mucronatus* in French Guiana [34]. In 2023, DNA from two *Bartonella* species was identified in *T. rubrofasciata* in Guangdong Province, China [35]. Additionally, *B. henselae* DNA was found in *Triatoma sordida* specimens collected near residential areas in Brazil [36]. Nevertheless, it remains unclear whether *Bartonella* can replicate and proliferate within triatomines, or whether these insects are capable of transmitting *Bartonella* to humans and animals.

This study aimed to identify *Bartonella* species in *T. rubrofasciata*, a common ectoparasite in southern China that frequently bites humans. Additionally, the study assessed the potential role of *T. rubrofasciata* as a vector for *Bartonella spp.* transmission to humans and animals.

## Materials and methods

### Ethical considerations

All animal procedures and experiments adhered to the ethical guidelines for the Care and Use of Laboratory Animals in China, with approval from the Guangxi Medical University Ethics Committee (grant number 82260413).

### Sample collection and species identification

Samples of triatomines, fleas, ticks, and rats were gathered from various cities in the Guangxi Zhuang Autonomous Region and Hainan Province, Southern China. Triatomine samples from Guangxi were collected between May and October annually from 2021 to 2023, while those from Hainan were obtained in November 2024. Fleas, ticks, and rats were gathered between June and September 2023. Fleas were sourced from the Nanning Animal Shelter. Ticks were collected from goats, cattle, and pets in the cities of Liuzhou, Hechi, and Baise, Guangxi, while wild rats were collected from Yizhou Prefecture, Hechi City, Guangxi. All samples were transported to the Parasitology Laboratory at Guangxi Medical University in Nanning, where they were morphologically identified according to established protocols [37-40].

### DNA extraction and polymerase chain reaction (PCR) detection

Triatomine, tick, and flea samples were sequentially cleaned three times in 70% ethanol and PBS solution at room temperature. For triatomines, the intestines and heads, containing the salivary glands, were dissected and separated. Ticks were bisected with sterile blades. The heads of the triatomines, ticks, and individual fleas were then placed in PBS solution for DNA extraction. Wild rats were anesthetized by intraperitoneal injection of a ketamine-xylazine solution (100 mg/kg ketamine + 50 mg/kg xylazine), euthanized through cervical dislocation, and their spleens and hearts were dissected for DNA extraction. Whole-genome DNA was extracted using a genomic DNA extraction kit (TIANGEN, Beijing, China) according to the manufacturer's protocol.

*Bartonella*-specific genes (*gltA*, *ITS*, and *rpoB*) were amplified using the following primers: BhCS.781p and BhCS.1137n for *gltA* [41], Ba325s and Ba1100as for *ITS* [42], and rpoBF and rpoBR for *rpoB* [43] (primer details in Supplementary Table S1). PCR reactions were conducted in a 25 μl volume containing 30 ng of DNA template, 1× Taq PCR Master Mix (Takara, China), and 0.4 μM of each primer. Thermal cycling conditions were as follows: initial denaturation at 95°C for 3 min; 35 cycles of denaturation at 94°C for 30 s, annealing at 55-58°C for 60 s, and extension at 72°C for 90 s, followed by final extension at 72°C for 10 min. Amplified products were purified with the HiPure Gel Pure DNA Mini Kit (Magen, China) and bi-directionally sequenced by Sangon Biotech (Shanghai, China).

### Molecular and phylogenetic analyses

Sequences obtained were compared to those available in the GenBank database using the BLAST algorithm (http://blast.ncbi.nlm.nih.gov/Blast.cgi) to identify homologous sequences and retrieve data from various *Bartonella* species. Discrepancies in species identification across the three gene primers (*gltA*, *ITS*, and *rpoB*) prompted further PCR analysis using primers targeting the *nuoG* gene (nuoGF and nuoGR) [44] and the *ribC* gene (BARTON-1 and BARTON-2) [45] (primer details in Supplementary Table S1). Multiple sequence alignments of homologous *Bartonella* from different hosts and geographic regions were downloaded from the NCBI database (sequence details in Supplementary Table S2) and aligned with ClustalW (https://www.genome.jp/tools-bin/clustalw). Phylogenetic trees were constructed using maximum-likelihood and neighbour-joining models based on the *gltA* and *ITS* genes in MEGA 11, with bootstrap support of 1000 replicates.

### Duration of bartonella detection in triatomine feces

PCR detection of fecal DNA from triatomines confirmed infection with *B. rochalimae* in two triatomines and *B. elizabethae* in one. The insects were housed individually in 12 cm × 8 cm × 6 cm semi-closed cubic containers. Fresh triatomine feces were collected weekly over eight weeks and stored at – 80 °C until DNA extraction. Fecal DNA extraction and *ITS* sequence amplification of *Bartonella* were carried out according to previously established methods.

### Transovarian transmission experiment

To assess the potential for transovarial transmission of *Bartonella* in *T. rubrofasciata*, eggs from *B. rochalimae* and *B. elizabethae*-infected *T. rubrofasciata* were hatched and reared in 12 cm × 8 cm × 6 cm semi-closed cubic containers with nylon gauze. First-stage nymphs were provided blood from C57BL/6J mice throughout their nymphal life cycle, until reaching adulthood. Fresh fecal samples were collected from nymphs (stages III and IV) and adults. As described previously, DNA extraction followed by *ITS* sequence PCR amplification was employed to detect *Bartonella* in eggs, stages I and II nymphs, and the feces of stages III, IV, and V nymphs and adults.

### Infection experiment

To assess the potential of *T. rubrofasciata* as a vector for *Bartonella* transmission, *B. elizabethae* DNA was detected in the fresh feces of two wild-caught triatomines via PCR. Each infected *T. rubrofasciata* was individually housed and fed in a semi-closed cubic container (12 cm × 8 cm × 6 cm). Weekly, the triatomines fed on C57BL/6J mice for approximately 30 min per session. Fresh feces excreted by the triatomines were collected, mixed with 100 µl PBS, and injected intraperitoneally into mice. Blood samples from the bitten and blood-fed mice, as well as from those injected intraperitoneally, were collected via tail vein on days 3, 7, 14, and 21 post-infection to test for *B. elizabethae* presence. Infected mice were subsequently exposed to laboratory-bred *T. rubrofasciata*. Fresh feces from these bugs were also collected and analyzed for *B. elizabethae* as previously described.

## Results

### Specimen identification

**Figure 1.**
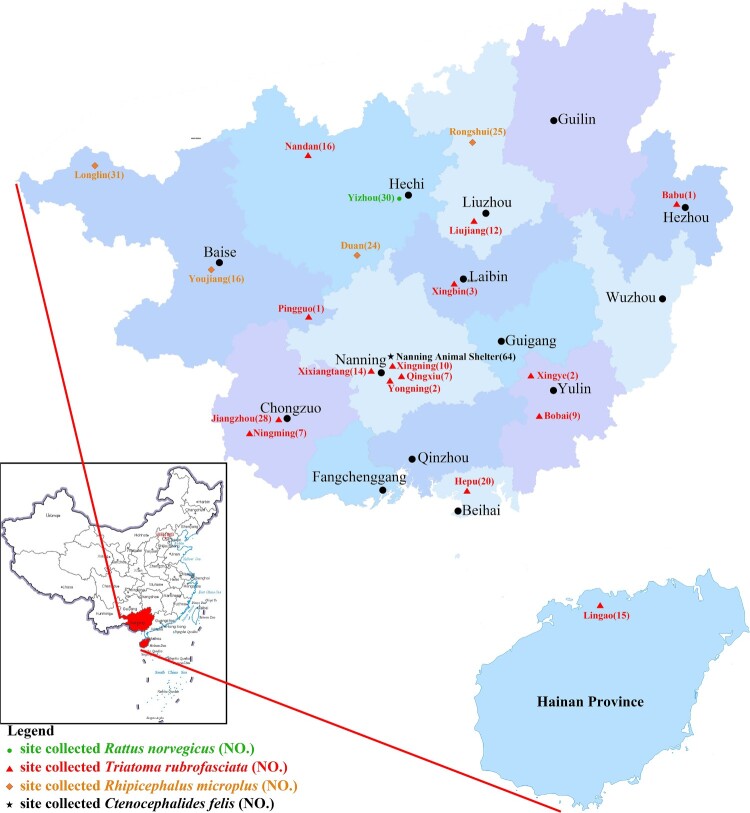
**Collection sites and numbers of *Triatoma rubrofasciata*, Ctenocephalides felis, Rattus norvegicus, and Rhipicephalus microplus in Guangxi and Hainan**. Triangles indicate the collection sites of *Triatoma rubrofasciata*, dots represent Rattus norvegicus collection sites, stars denote Ctenocephalides felis collection sites, and rhombuses represent Rhipicephalus microplus collection sites*.*

A total of 147 triatomines were collected from ten cities in Guangxi and Hainan provinces: Beihai, Chongzuo, Yulin, Nanning, Laibin, Baise, Hechi, Liuzhou, Hezhou, and Lingao. All specimens were identified as *T. rubrofasciata* through morphological analysis [37]. In Yizhou, Hechi City, 30 wild rats were captured and classified as *Rattus norvegicus* based on morphological traits [40]. Additionally, 96 ticks were collected from Liuzhou, Hechi, and Baise; 82 were from cattle and buffalo farms, while 14 were obtained from pets. These ticks were identified as *Rhipicephalus microplus* using morpho-taxonomic keys [39]. Furthermore, 64 fleas were collected from ten cats and two dogs at the Nanning Animal Shelter and morphologically identified as *Ctenocephalides felis* [38]. Collection sites and the number of *T. rubrofasciata*, *R. norvegicus*, *R. microplus*, and *C. felis* were illustrated in Figure 1.

### Prevalence and species of Bartonella in T. rubrofasciata

Table 1 presents the prevalence of *Bartonella* and its species in triatomines, fleas, rats, and ticks. *Bartonella* was detected in 25 of 147 triatomine samples, corresponding to a 17.01% positivity rate. Among the samples from Guangxi, the positivity rate was 16.67% (22/132), while in Hainan, it was 20% (3/15). All positive samples were identified in intestinal DNA, with five also yielding DNA from the heads (Table 1). *Bartonella* infection was observed in both adult and nymph stages. Co-infection in two samples resulted in the recovery of 27 sequences, producing fragments of 379, 850, and 580 bp for the *gltA*, *rpoB*, and *ITS* genes, respectively. BLASTn analysis of the *ITS* and *gltA* sequences from these strains revealed diverse *Bartonella* species: six strains aligned more closely with *B. queenslandensis* (accession no. EU111769 and MZ570397), showing nucleotide identities of 96.10% – 97.38%; three strains exhibited higher homology with *B. silvatica* (accession no. AB498008), with nucleotide identities of 94.41% – 94.67%; one strain was closely related to *B. coopersplainsensis* (accession no. MK562490), with a nucleotide identity of 95.71%; thirteen strains aligned with *B. rochalimae* (accession no. DQ683199 and DQ676487), with nucleotide identities ranging from 95.34% to 99.27%; two strains showed higher similarity to *B. elizabethae* (accession no. LR746190), with nucleotide identities of 97.22% and 98.91%; and two strains were closely related to *B. tribocorum* (accession no. LR746190), with nucleotide identities of 98.85% and 99.72%. Details of the amplification of the *Bartonella gltA*, *ITS*, and *rpoB* genes, as well as the specific identification of *T. rubrofasciata*, were provided in Supplementary Table S3.

**Table 1.** The *Bartonella* species identification in *Triatoma rubrofasciata*, *Ctenocephalides felis*, *Rattus norvegicus* and *Rhipicephalus microplus*.

Sample Species	Collected No.	Poscitve No.	Identifed speices	Detected sites (No.)
		(ratio)		
Triatomine	147	25 (17.01%)	*B. rochalimae*	intestinal contents (13) heads (2)
			*B. coopersplainsensis*	intestinal contents (1) heads (0)
			*B. silvatica*	intestinal contents (3) heads (1)
			*B. queenslandensis*	intestinal contents (6) heads (1)
			*B. elizabethae*	intestinal contents (2) heads (1)
			*B. tribocorum*	intestinal contents (2) heads (0)
Flea	64	60 (93.75%)	*B. rochalimae*	
			*B. clarridgeiae*	
			*B. henselae*	
Tick	96	5 (5.21%)	*B. queenslandensis*	
			*Bartonella sp*.	
rat	30	7 (23.33%)	*B. queenslandensis*	spleen (6) kidney (5)
			*B. silvatica*	spleen (1) kidney (1)
Total	337	97 (28.78%)		

### Prevalence and species of Bartonella in fleas, ticks, and rats

*Bartonella* was detected in 60 of 64 flea samples (93.75% infection rate) via PCR amplification targeting the *ITS*, *gltA*, and *ropB* genes. At least two samples from each cat and dog were sequenced, and discrepancies in species identification between these genes prompted additional PCR testing. Among the 26 samples sent for sequencing, one corresponded to *B. rochalimae* (100% nucleotide identity; accession number DQ676491), previously detected in dogs in the USA. Another matched *B. henselae* (99.81% nucleotide identity; accession number CP072898) was found in Felis catus in Germany. Twenty-four samples were co-infected with *B. clarridgeiae* and *B. henselae*. The highest homology with *B. clarridgeiae* (97.82% – 99.81% nucleotide identity; accession numbers CP116497 and OK624793) was observed in fleas from *C. orientis* in Malaysia and *Felis catus* in Spain. Similarly, the highest homology with *B. henselae* (99.65% – 99.88% nucleotide identity; accession numbers CP072898 and CP020742) was found in samples from *Felis catus* and Homo sapiens in Germany. In rats, PCR amplification of the *ITS* and *gltA* genes detected *Bartonella* in 7 of 30 samples (23.33%). DNA sequencing and BLASTn comparison of the *gltA* (379 bp) and *ITS* (580 bp) genes showed that six samples were most closely related to *B. queenslandensis* (96.12–98.24% nucleotide identity; accession numbers MZ570397 and MH748120). One sample aligned with *B. silvatica* (accession number AB498008), isolated from *Rattus fulvescens* in China. In ticks, 5 of 96 samples (5.21%) tested positive for *Bartonella* via *gltA* amplification and sequencing. Three sequences exhibited the highest homology (97.64% – 98.22%) with *B. queenslandensis* (accession number MH748120), isolated from *Niviventer confucianus* in China. The remaining two sequences matched an unidentified *Bartonella* species (98.04% and 99.03% nucleotide identity; accession number KX000252) detected in hard ticks from Tibet, China. Details on the amplification of *Bartonella gltA*, *ITS*, and *rpoB* genes, as well as the identification of *C. felis*, *R. microplus*, and *R. norvegicus*, were provided in Supplementary Table S3.

### Phylogenies based on gltA and ITS sequences

**Figure 2.**
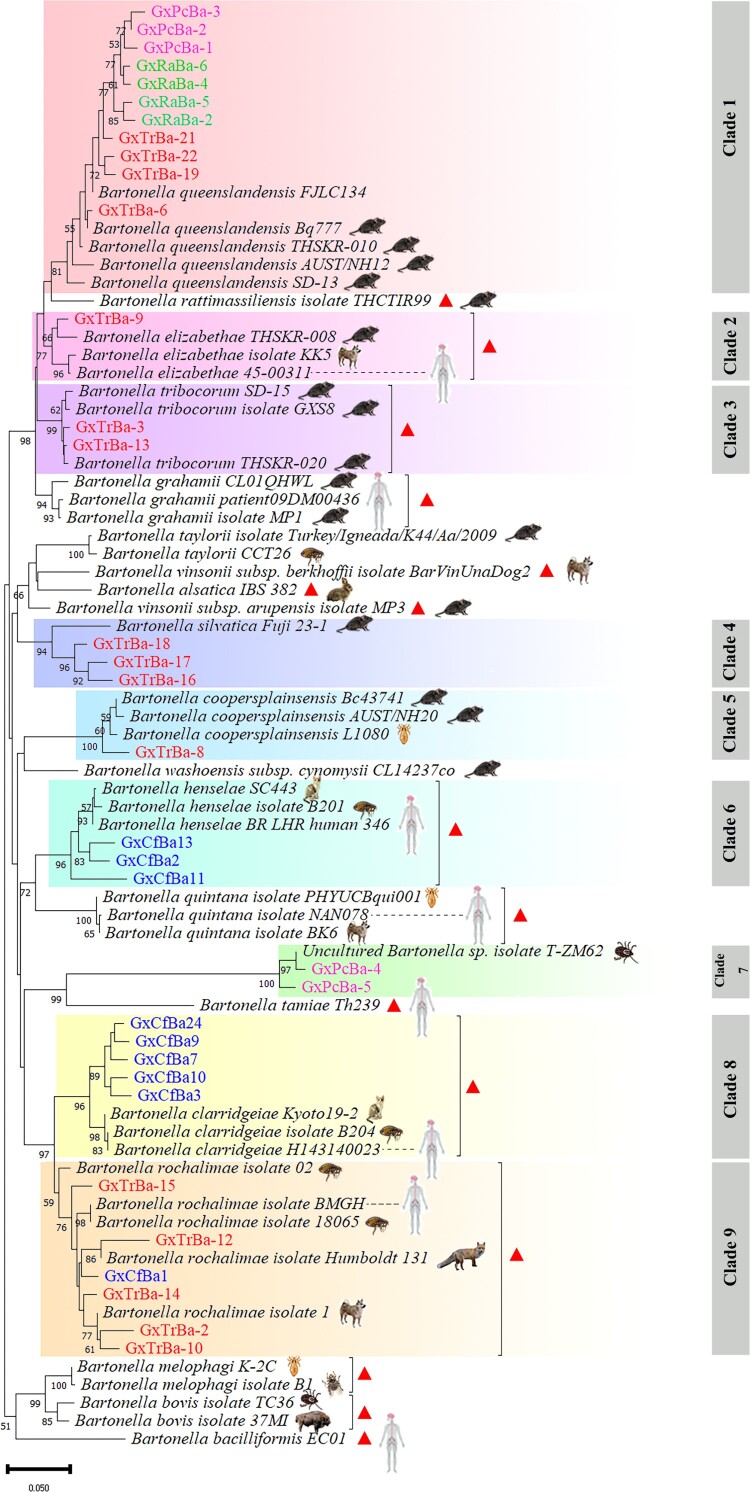
**Phylogenetic tree of *Bartonella spp.* based on the *gltA* gene**. *Bartonella* sequences obtained from *Triatoma rubrofasciata*, *Ctenocephalides felis*, *Rattus norvegicus*, and *Rhipicephalus microplus* are highlighted in red, blue, green, and purple text, respectively. The phylogenetic tree was constructed using the neighbor-joining (NJ) method in MEGA, with 1000 bootstrap replications. The accession numbers for the *gltA* gene sequences of *Bartonella spp.* are listed in Supplementary Table S2. Host species for reference sequences are indicated by icons, and *Bartonella* species capable of infecting humans are marked with red triangles. The phylogenetic analysis revealed the isolation of *Bartonella* into nine distinct clades. *Bartonella* sequences from *T. rubrofasciata* are grouped into Clades 1, 2, 3, 4, 5, and 9.

**Figure 3.**
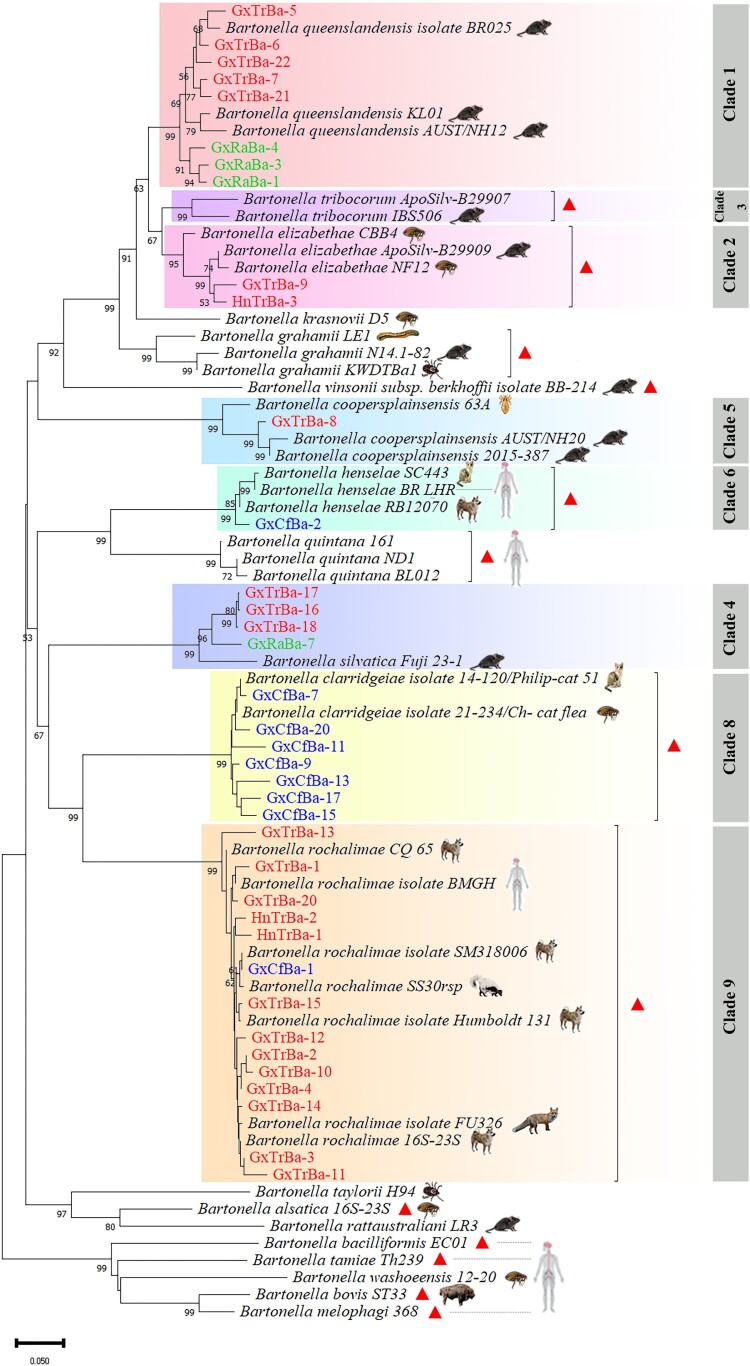
**Phylogenetic tree of *Bartonella spp.* based on the *ITS* gene.** *Bartonella* sequences from *Triatoma rubrofasciata*, *Ctenocephalides felis*, *Rattus* norvegicus, and Rhipicephalus microplus are highlighted in red, blue, green, and purple, respectively. The phylogenetic tree was constructed using the neighbor-joining (NJ) method in MEGA, with 1000 bootstrap replications. Accession numbers for the *ITS* sequences of *Bartonella spp.* are listed in Supplementary Table S2. Icons denote the host species corresponding to the reference sequences, and *Bartonella* species capable of infecting humans are marked with red triangles. Phylogenetic analysis revealed that the isolated *Bartonella* sequences clustered into nine distinct clades. Sequences from *T. rubrofasciata* were assigned to Clades 1, 2, 4, 5, and 9.

The *gltA* and *ITS* sequences obtained were used to construct phylogenetic trees, incorporating representative species from all major *Bartonella* clades (Figures 2 and 3). Phylogenetic analysis revealed distinct clustering patterns among isolates from different host species. The *gltA* phylogenetic tree included 34 *Bartonella* sequences from this study and homologous sequences distributed across nine clades. Sixteen *T. rubrofasciata* isolates were grouped into six clades: Clades 1, 2, 3, 4, 5, and 9 (Figure 2). These clustered with *B. queenslandensis* (from rats), *B. elizabethae* (from humans, rats, and dogs), *B. tribocorum* (from rats), *B. silvatica* (from Rattus), *B. coopersplainsensis* (from Rattus and lice), and *B. rochalimae* (from humans, foxes, dogs, and fleas). Nine flea isolates were distributed among three clades: Clades 6, 8, and 9 (Figure 2), clustering with *B. henselae* (from humans, cats, and fleas), *B. clarridgeiae* (from cats, fleas, and humans), and *B. rochalimae*. Five tick isolates were distributed between two clades, Clades 1 and 7 (Figure 2), and clustered with *B. queenslandensis* and *Bartonella* sp. (from ticks). Four rat isolates formed a single clade, Clade 1, which clustered with *B. queenslandensis* (Figure 2). Overall, *T. rubrofasciata* harbored a higher number of *Bartonella* species compared to ticks, fleas, and wild rodents and shared species with these vectors and hosts.

The *ITS* sequence phylogenetic tree included 37 *Bartonella ITS* sequences and homologous sequences, categorized into seven distinct clades. Of the 24 isolates from *T. rubrofasciata*, five clades – Clades 1, 2, 4, 5, and 9 – were represented (Figure 3). These isolates clustered with *B. queenslandensis* (from rats), *B. elizabethae* (from *Leptopsylla taschenbergi* and *Rattus*), *B. silvatica* (from *Rattus*), *B. coopersplainsensis* (from *Rattus* and fleas), and *B. rochalimae* (from humans, foxes, and dogs). The nine isolates from fleas were distributed across three clades – Clades 6, 8, and 9 (Figure 3) – and clustered with *B. henselae* (from humans, cats, and dogs), *B. clarridgeiae* (from cats and fleas), and *B. rochalimae*. Four isolates from rats were assigned to Clades 1 and 4, clustering with *B. queenslandensis* and *B. silvatica* (Figure 3)*.*

### Duration of Bartonella detection in triatomine feces

*ITS* gene amplification detected *Bartonella* in feces collected at various stages. PCR analysis identified both *B. rochalimae* and *B. elizabethae*, two zoonotic species, in samples collected on five consecutive occasions. Remarkably, *Bartonella* remained detectable in the feces of wild triatomines, even after eight weeks of rearing (Supplementary Figure S1).

### Experimental transmission of Bartonella

Experimental infection studies have shown that *B. elizabethae* can be detected in the blood of C57BL/6J mice 14 days post-inoculation with feces containing *Bartonella*, administered either via intraperitoneal injection or through a triatomine bite, as confirmed by *ITS* PCR amplification. Remarkably, infected mice did not display typical symptoms such as anemia or depression. Laboratory-reared *T. rubrofasciata* did not show detectable levels of *B. elizabethae* in their feces when tested by PCR 21 days after feeding on infected mice. However, when fecal samples were mixed with Dulbecco's Modified Eagle’s medium (DMEM) and incubated under enriched culture conditions at 37°C with 5% CO_2_ for 12 days [46], PCR testing identified *B. elizabethae*. Transovarial transmission experiments showed that nymphs from a subsequent generation of three *T. rubrofasciata* infected with *B. rochalimae* and two infected with *B. elizabethae*, which were fed for 20 weeks until adulthood, exhibited no detectable *Bartonella* in eggs, stages I and II nymphs, or fresh feces during the entire period.

## Discussion

**Figure 4.**
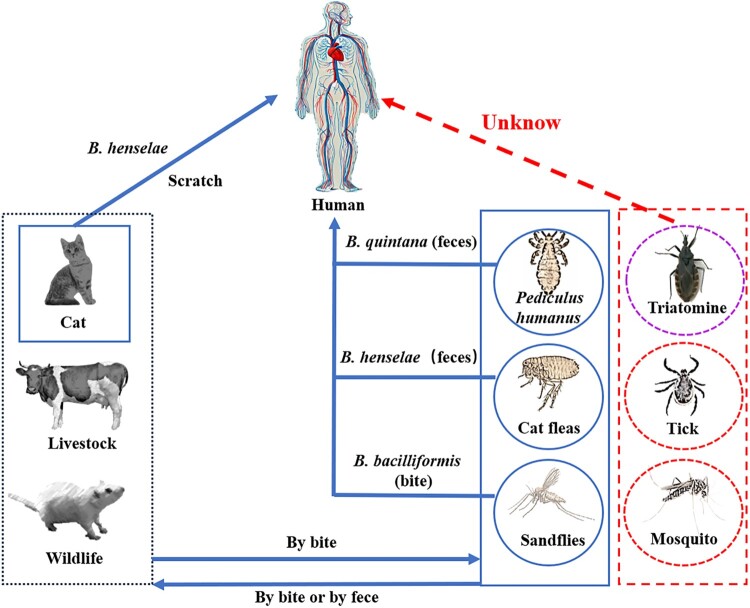
**Transmission routes of *Bartonella* among human, mammal, and arthropod vectors.** *Bartonella* primarily spreads to humans via arthropod vector bites or through fecal contamination of wounds. Known vectors of *Bartonella* include fleas, lice, and sandflies, while other potential vectors, such as ticks, mites, mosquitoes, and triatomines, have been shown to harbor *Bartonella*, though their role in human transmission remains uncertain. Cats and dogs may transmit *Bartonella* to humans through scratches and bites, but the precise transmission mechanism is still unclear. The blue line indicates confirmed *Bartonella* transmission routes, while the dashed line represents potential, but unconfirmed, routes.

Bartonellosis is a serious global zoonotic disease, yet the transmission pathways of *Bartonella* infections in humans remain incompletely understood. Arthropod-borne transmission is regarded as the important route for human bartonellosis [47]. However, the specific vectors and transmission pathways for human bartonellosis are still not fully clarified. The cat flea plays a crucial role in the transmission cycle of *B. henselae*. These fleas acquire, maintain, and spread the bacterium among cats by feeding on their blood [33]. Contaminated flea feces can enter human skin wounds or mucous membranes, often through cat scratches or bites, thereby facilitating bacterial inoculation [48,49]. Body lice transmit *B. quintana* to humans through fecal contamination of open wounds [50], while sandflies (*Lutzomyia verrucarum*) spread *B. bacilliformis* via bites [16]. Additional vectors, including mites, mosquitoes, and ticks, have been shown to harbour *Bartonella* spp [26-28], but no strong evidence currently supports their role in transmitting *Bartonella* to humans. In addition to vector transmission, direct contact may also contribute to infection. A case report documented human infection with *B. vinsonii subsp. berkhoffii* following accidental needle puncture with a contaminated needle [51]. Moreover, *B. henselae* has been detected in the saliva of cats and dogs [52,53], suggesting potential direct transmission from infected animals to humans. In summary, our understanding of the transmission mechanisms of human *Bartonella* remains incomplete (Figure 4), presenting substantial challenges for effective prevention and control of bartonellosis.

Six *Bartonella* species were identified in *T. rubrofasciata* for the first time, including *B. rochalimae*, *B. coopersplainsensis*, *B. silvatica*, *B. queenslandensis*, *B. elizabethae*, and *B. tribocorum*. Notably, *B. rochalimae*, *B. tribocorum*, and *B. elizabethae* are known to infect humans. *Bartonella rochalimae* has been detected in various regions across the globe [18],[54-57], but its transmission route remains unclear; some human cases have been linked to contact with dogs [18]. The species has a broad range of natural hosts, including rodents (54), cats, dogs (55), foxes (56), and wolves [57], and has also been detected in arthropods such as fleas [54] and ticks [58]. In this study, *B. rochalimae* was found to have a high infection rate (8.84%, 13/147) in *T. rubrofasciata*, detected in both intestinal contents and heads (containing salivary glands). This species was also identified in cat fleas, with sequence clustering matching those from *T. rubrofasciata* (Figure 2, 3). Additionally, *B. rochalimae* was shown to persist in *T. rubrofasciata* for extended periods. These results suggest that, although human *B. rochalimae* infections via insect bites have not been reported, the possibility of vector transmission cannot be ruled out.

*Bartonella elizabethae* and *Bartonella tribocorum*, both zoonotic species, were identified in *T. rubrofasciata*. *B. elizabethae* is known to cause endocarditis and neuroretinitis in humans [59], and has been detected in febrile patients [59], HIV-infected individuals [17], and heroin addicts [60], with the latter group showing a notably high seropositivity rate of 39%. Known hosts of *B. elizabethae* include rodents (*R. rattus*, *R. norvegicus*, and *Acomys cahirinus*) [61,62] and fleas (*C. felis* and *Xenopsylla sp.*) [63,64]. McKee et al. [64] demonstrated that *Xenopsylla cheopis* could harbour *B. elizabethae* for at least 13 days post-blood meal, indicating the potential role of blood-feeding arthropods in transmitting these *Bartonella* species between rodents and humans. Furthermore, human infection with *B. elizabethae* has been linked to contact with dogs and tick bites [19]. *B. tribocorum*, genetically similar to *B. elizabethae*, causes lymphadenopathy in humans and has been found in vectors such as fleas [65], lice [66], and ticks [67]. In this study, both *B. elizabethae* and *B. tribocorum* were exclusively detected in *T. rubrofasciata*, with no presence in cat fleas, rats, or ticks. This may be attributed to the flight capability of *T. rubrofasciata*, which allows it to feed on a broader range of hosts, potentially harboring a wider diversity of *Bartonella* species compared to more restricted vectors like fleas and ticks*.*

*Triatoma rubrofasciata*, a vector of *T. cruzi* with a global distribution, transmits pathogens through feces and bites [68]. Its habitat overlaps with those of humans and livestock, resulting in occasional human bites. Previous studies have confirmed the widespread presence of *T. rubrofasciata* in southern China, with a rising incidence of triatomine bites [37]. Our unpublished data suggests that the primary blood hosts for Triatomines in Guangxi, China, include rats, cattle, pigs, chickens, birds, and humans. This broad spectrum of blood hosts enhances the likelihood of pathogen transmission between animals and humans. In this study, three zoonotic *Bartonella* species were identified in the salivary glands and gut of *T. rubrofasciata*. Additionally, *Bartonella* was found to persist in *T. rubrofasciata* for extended periods. Laboratory experiments further demonstrated that *Bartonella* can infect mice through intraperitoneal injection of triatomine feces and via triatomine bites. These results indicate that *T. rubrofasciata* may serve as a potential vector for *Bartonella*, despite the absence of transovarial transmission. However, there is a lack of clinical evidence to support triatomine-mediated transmission and its causation of human bartonellosis. This evidence gap is likely due to the nocturnal activity of triatomines, which rest during daylight hours. The inconspicuous nature of triatomine bites often leads to insufficient awareness among both patients and clinicians regarding the potential risks of these bites and the associated transmission of pathogens causing various diseases.

Pathogens such as *T. cruzi* can reproduce and transmit through triatomines, which must also endure immune responses from the vector [69]. The gut microbiota of triatomines may influence this process significantly [70]. For instance, *Rhodococcus*, a key player in the metabolism of *T. rubrofasciata*, has been demonstrated to effectively eliminate pathogens [71]. Furthermore, the pathogen's ability to replicate in the triatomine midgut is essential for transmission. This study identified *B. elizabethae* and *B. rochalimae* consistently in the feces of *T. rubrofasciata*, with *Bartonella* also detectable in the heads of triatomines. These findings suggest that *Bartonella* can resist the immune response of *T. rubrofasciata*, proliferate, and migrate to the salivary glands, thereby enabling pathogen transmission.

To investigate the carriage of *Bartonella* species by various vectors and their interrelationships, a comprehensive analysis was conducted on fleas, ticks, rodents, and *T. rubrofasciata*. *B. queenslandensis* was detected across all four vector types, while *B. rochalimae* was found in both *T. rubrofasciata* and fleas, and *B. silvatica* was identified in both rodents and *T. rubrofasciata*. Species exclusive to specific vectors included *B. henselae* and *B. clarridgeiae*, detected only in fleas, and *B. coopersplainsensis*, *B. elizabethae*, and *B. tribocorum*, which were restricted to *T. rubrofasciata*. These results indicate that *T. rubrofasciata* shares several *Bartonella* species with other vectors and reservoir hosts, while also harboring a broader range of *Bartonella* species than the other vectors studied.

PCR amplification and sequence analysis of the *ITS* and *gltA* genes were employed to identify *Bartonella* species in *T. rubrofasciata*, *C. felis*, *R. microplus*, and *R. norvegicus*. In cases where amplification of *gltA* or *ITS* failed or yielded inconsistent results, additional genes (*rpoB*, *ribC*, or *nuoG*) were targeted to verify species identification using at least two distinct genes. Mixed infections with multiple *Bartonella* species were observed in triatomines and fleas, particularly in *C. felis*, where co-infection rates reached 92.30% for *B. clarridgeiae* and *B. henselae*. Detection of all species in mixed infections proved challenging when relying solely on *gltA* and *ITS* sequences, due to the amplification bias of certain *Bartonella* species identification genes, including *gltA*, *ITS*, and *rpoB*. This bias may result in incomplete detection of species present in mixed infections. In the *B. clarridgeiae* and *B. henselae* co-infection in fleas, the *ITS* gene preferentially amplified *B. clarridgeiae*, while *rpoB* favored *B. henselae*. The *gltA* gene was able to amplify both species, with priority identification potentially influenced by DNA concentration. In the *B. rochalimae* and *B. tribocorum* co-infection in *T. rubrofasciata*, the *ITS* gene exhibited a bias towards *B. rochalimae*, whereas *gltA* favored *B. tribocorum*. These results highlight the necessity of utilizing more than three genes for accurate species identification in mixed infections. Additionally, consistency across at least two genes provided more reliable confirmation of species identification.

Transovarial transmission of *Bartonella* varies among blood-feeding arthropods and between different *Bartonella* species. *B. washoensis* was detected in the ovaries of fleas collected from various mammals, supporting the potential for transovarial transmission [72]. Additionally, transovarial transmission of *B. henselae* and *B. quintana* has been observed in ticks (*Rhipicephalus sanguineus*) [73] and body lice [74], respectively. In contrast, *B. henselae* does not exhibit transovarial transmission in fleas [75], and *B. bacilliformis*, transmitted by sandflies, also lacks vertical transmission [76]. In this study, no transovarial transmission of *B. elizabethae* and *B. rochalimae*, carried by *T. rubrofasciata* was detected, suggesting that the primary source of *Bartonella* in *T. rubrofasciata* is likely the host and environment or horizontal transmission. However, due to the small sample size, further research is necessary to verify this conclusion.

The limitations of this study include the restricted sample size and geographic range of the collected vectors, particularly in the assessment of transovarial transmission. Additionally, no confirmed cases of *Bartonella* infection attributable to triatomine bites are identified in clinical settings, preventing clinical validation of triatomines as vectors for human bartonellosis. Nevertheless, the study suggests that *T. rubrofasciata* may serve as a potential vector for both human and animal bartonellosis, highlighting the need for heightened awareness of *Bartonella* transmission by triatomines, especially in regions where these insects are prevalent. Enhanced detection and surveillance of *Bartonella* in triatomines, animals, and humans, along with improved control measures, are essential to mitigate the spread of human bartonellosis. In future research, immunoscreening for *Bartonella* will be conducted in humans. Patients testing positive will be analyzed for associations with *T. rubrofasciata* bites. Whole-genome sequencing and traceability analysis of *Bartonella* will be performed in humans, *T. rubrofasciata*, and other vectors and hosts. In-vitro infection assays on erythrocytes and endothelial cells will be executed to determine whether *Bartonella* can infect human cells. These studies will provide clinical evidence for the potential role of *T. rubrofasciata* as a human *Bartonella* transmission vector.

## Conclusion

This study demonstrates that *T. rubrofasciata* can harbour multiple *Bartonella* species, including zoonotic strains, in both the salivary glands and the gut. Furthermore, *Bartonella* can persist within *T. rubrofasciata* for extended periods. Laboratory experiments confirm that *Bartonella* can infect mice via *T. rubrofasciata* bites and through intraperitoneal injection of triatomine feces. These results suggest that *T. rubrofasciata* is a potential vector for certain *Bartonella spp.*, despite the absence of transovarial transmission. This study provides new insights into the sources and mechanisms of *Bartonella* transmission, advancing our understanding of the role of triatomines as vectors.

## Author contributions

YLS, YWL, DYL, and PCD developed the study protocol. PCD, BLQ, ALL, QAZ, XYF, XQL, CHL, SHH, LLT, ZWZ, and WJC conducted the field and laboratory work and contributed to data analysis. YLS, DYL, YWL, and PCD performed the final data analysis. YLS and PCD drafted the manuscript. All authors reviewed and approved the final manuscript.

## Availability of data and materials

Data from this study are available upon request from the corresponding author.

## Supplementary Material

Supplementary Figure S1.tif

Supplementary Table S1.xlsx

Supplementary Table S3.xlsx

Supplementary Table S2.xlsx
