# Supplementary figures and images for: Triatoma rubrofasciata as a potential vector for bartonellosis
Source: Emerg Microbes Infect. 2025 Apr 15;14(1):2494291. doi: 10.1080/22221751.2025.2494291 (PMC12051607; doi:10.1080/22221751.2025.2494291)

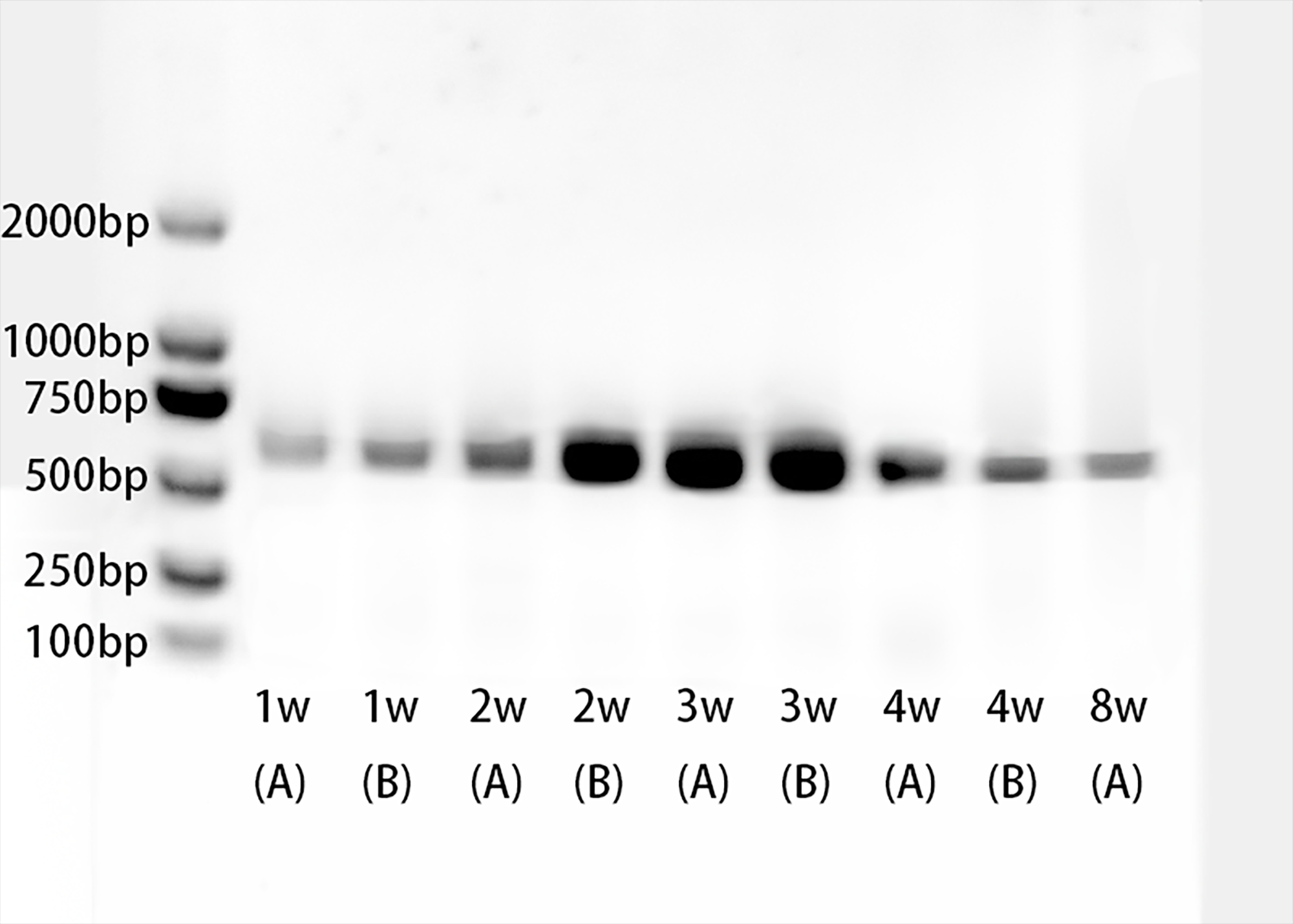

Supplement: Supplementary Figure S1.tif [file TEMI_A_2494291_SM2276.tif]
